# Oral and topical vitamin D treatment strategies in psoriasis

**DOI:** 10.1093/skinhd/vzaf010

**Published:** 2025-04-22

**Authors:** Michelle Mai, Ingrid Lazaridou, Fatima N Mirza, Karen H Costenbader, Abrar A Qureshi, Eunyoung Cho

**Affiliations:** Division of Dermatology, Warren Alpert Medical School of Brown University, Rhode Island Hospital, Providence, RI, USA; Division of Dermatology, Warren Alpert Medical School of Brown University, Rhode Island Hospital, Providence, RI, USA; Division of Dermatology, Warren Alpert Medical School of Brown University, Rhode Island Hospital, Providence, RI, USA; Division of Rheumatology, Inflammation and Immunity, Brigham and Women’s Hospital and Harvard Medical School, Boston, MA, USA; Division of Dermatology, Warren Alpert Medical School of Brown University, Rhode Island Hospital, Providence, RI, USA; Department of Epidemiology, Brown School of Public Health, Providence, RI, USA; Division of Dermatology, Warren Alpert Medical School of Brown University, Rhode Island Hospital, Providence, RI, USA; Department of Epidemiology, Brown School of Public Health, Providence, RI, USA; Channing Division of Network Medicine, Department of Medicine, Brigham and Women’s Hospital and Harvard Medical School, Boston, MA, USA

## Abstract

Psoriasis is a prevalent skin disorder affecting approximately 2–3% of the population in the USA. Its complex and varied presentations necessitate a diverse range of available therapeutic options. While topical corticosteroid therapy is conventionally employed as first-line treatment, long-term usage increases the risk of adverse events, prompting the consideration of alternatives including steroid-sparing agents such as vitamin D. In this article, we review literature from topical and oral vitamin D trials for the treatment of psoriasis. Topically, vitamin D analogues have been well established as an effective long-term treatment, particularly when used in combination with other therapies. Moreover, combination therapy with immunomodulators such as apremilast and methotrexate has shown promise as well. Conversely, oral vitamin D supplementation trials have yielded more inconsistent results, with some supplementation clinical trials showing significant psoriasis resolution and others showing no significant changes in psoriasis outcome. Vitamin D deficiency status, seasonal variation and body mass index were factors that may have modulated the therapeutic effect of vitamin D supplementation. Further study combining vitamin D supplementation with pre-existing treatments may also augment the effect of monotherapy. Studies on the synergistic effects of combination therapies with oral vitamin D or the development of foam-based or microneedle drug delivery systems may be promising next steps.

## Teaser

Emerging evidence shows potential benefits of vitamin D supplementation, especially regarding combination therapies and usage among patients with low circulating vitamin D levels. This review summarizes the clinical benefit of vitamin D treatments in psoriasis and proposes directions for future research to improve treatment options and outcomes.

## Introduction

Psoriasis, a papulosquamous skin disorder, manifests in a spectrum of clinical phenotypes, but in most classic forms presents at times as coalescent erythematous papules and plaques with micaceous scale.^1^ Approximately 2–3% of the population of the USA carries a diagnosis of psoriasis.^2–4^ The pathogenesis of psoriasis is multifactorial, involving genetic predispositions that may be exacerbated by environmental triggers, as well as intricate immunological processes that position psoriasis as both a chronic and systemic disease.^5–7^ Given the disease’s complexity and varied natural history, treatment strategies are diverse, ranging from limited skin-directed therapies to oral, intramuscular or intravenous systemics. Treatment regimens can be tailored to individual needs, factoring in disease severity and related comorbidities.^8^ Recent research has highlighted vitamin D’s role in regulating immune function, cell proliferation, differentiation and apoptosis,^9^ with the active form being involved with keratinocyte response;^10^ this work has renewed interest in the potential for vitamin D to play a more pivotal role in the psoriatic treatment ladder.

Vitamin D, a fat-soluble vitamin, is acquired through dietary intake of certain foods and is synthesized in the skin upon Ultraviolet (UV) exposure.^11,12^ It undergoes biotransformation via two hydroxylation reactions in the liver and kidney to produce 25(OH)D (25-hydroxyvitamin D, i.e. calcidiol) and 1,25(OH)_2_D (i.e. calcitriol). The biologically active form, calcitriol, is crucial for maintaining calcium absorption homeostasis and preserving the skeletal integrity. Calcipotriol (calcipotriene) is a synthetic drug derived from calcitriol. Given vitamin D’s integral role in regulating immune regulation and skin physiology, numerous clinical trials have been conducted to assess its therapeutic potential in psoriasis. Previous reviews explored nutritional components of oral vitamin D in psoriasis, but this review aims to comprehensively discuss the promising and prospective treatment strategies regarding both oral and topical vitamin D for psoriasis.^13–16^

## Methods

### Search strategy

A systematic search was conducted using the PubMed and Embase databases, covering literature from 1980 up until June 2023. The search utilized the following search strategy: ‘vitamin D’ OR ‘calcipotriol’ OR ‘calcipotriene’ OR ‘calcitriol’ OR ‘oral vitamin D’ OR ‘cholecalciferol,’ AND with ‘psoriasis’ AND ‘treatment’. This was designed to aggregate studies examining the impact of both topical and oral vitamin D on psoriasis treatment outcomes.

### Study selection

Criteria for inclusion encompassed full-text, English-language, peer-reviewed original research articles. The scope was narrowed to clinical trials that specifically evaluated the effectiveness or risk profile of vitamin D treatment, or its use in combination with other treatments, for psoriasis. Exclusion criteria applied to studies that either did not focus on vitamin D treatment for psoriasis or had a study size of fewer than five patients. There were no limitations regarding participant age, psoriasis subtype, severity of disease or specific form of vitamin D treatment (e.g. drug formulations, chemical nomenclature etc.). Studies on biological mechanisms of vitamin D in psoriasis were chosen based on full-text, English-language, peer-reviewed articles. Original research and reviews were included. No limitations on study model, psoriasis subtype or vitamin D formulation were made.

## Results

### The effects of vitamin D on skin cells

Vitamin D plays a multifactorial role in skin physiology, being directly synthesized in keratinocytes upon UVB radiation exposure.^12,17,18^ This initiates a photochemical reaction of 7-dehydrocholesterol (7DHC) to prohormone D3, ­culminating in the production of the biologically active 1,25-­dihydroxyvitamin D3. This active form interacts with vitamin D receptors (VDRs) in keratinocytes, orchestrating regulation of growth and differentiation pathways.^19–22^  *In vitro* studies have provided evidence of this direct influence; VDR knockout mice exhibit diminished levels of proteins essential for skin maturation, such as involucrin, profilaggrin and loricrin, which are pivotal for the cornified envelope’s development.^23,24^ The underlying biochemical mechanisms involve a cascade regulated by calcium-­mediated interactions between the vitamin D receptor and proteins such as the vitamin D receptor-interacting protein (DRIP) and steroid receptor coactivator (SRC).^25,26^ This regulatory interaction underscores vitamin D’s critical role in keratinocyte differentiation, which plays an integral role in the pathogenesis of psoriasis as well.

### Role of vitamin D in regulating the immune system

Vitamin D functions as an immunomodulator,^27^ with its active metabolites engaging VDRs to influence T-cell behaviour, including activation, proliferation and metabolism.^28^  *In vitro* studies of human CD4^+^ cells show that T-cell receptor signalling modulates VDR activation, which is further stabilized by the binding of vitamin D3.^29^ Activated VDRs modulate T-cell activities, downregulating certain T-cell activations and augmenting regulatory T-cell pathways.^30–32^ This modulation results in the inhibition of inflammatory T helper (Th) 1 and Th17 cellular differentiation and their cytokine production, while simultaneously promoting Treg pathways characterized by anti-inflammatory makers like interleukin (IL)-10, transforming growth factor (TGF)-β, cytotoxic T-lymphocyte associated protein 4 (CTLA-4) and CD25.

### Mechanism of action of vitamin D in psoriasis

**Figure 1:**
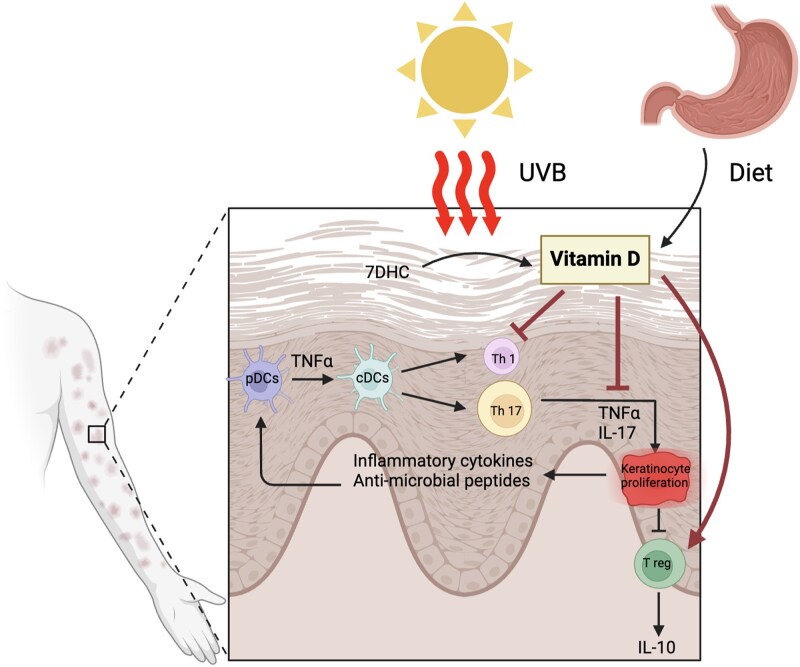
Mechanism of action for vitamin D in psoriasis. 7DHC, 7-dehydrocholesterol; IL-17, interleukin 17; pDC, plasmacytoid dendritic cell; Th1, T helper 1; Th17, T helper 17; TNFa, tumor necrosis factor-alpha; UVB, ultraviolet B.

Psoriasis, characterized by chronic inflammation and keratinocyte proliferation, involves intricate autoinflammatory and T-cell-mediated autoimmune responses.^33^ Autoinflammatory pathways lead to increased infiltration of effectors such as plasmacytoid dendritic cells (pDCs), myeloid dendritic cells (CD11c^+^ mDCs), neutrophils and natural killer (NK) cells, and antimicrobial peptides (AMPs).^34–37^ The downstream effects of these increased effectors lead to activated immune pathways, eventually increasing keratinocyte proliferation and sustained inflammation. Concurrently, the T-cell-driven autoimmune reactions involve activation of CD4^+^ Th1 and CD8^+^ cytotoxic T cells,^38–41^ leading to release of cytokines like IL-23, IL-17, interferon (IFN)-γ and tumour necrosis factor (TNF)-α, furthering the inflammatory loop.^42,43^ An adult human keratinocyte model showed that vitamin D, interacting with VDRs in keratinocytes, exhibits anti-inflammatory and antiproliferative properties through G2/M arrest.^44^ By modulating proinflammatory cytokines and T-cell responses, vitamin D works to restrain the activated immune pathways and curb the excessive proliferation characteristic of psoriatic lesions (Figure 1).^45,46^ Thus, vitamin D’s ability to suppress inflammatory and immune-­mediated responses may be a significant aspect of its therapeutic potential in psoriasis.

### Topical vitamin D treatments

The American Academy of Dermatology (AAD) and the National Psoriasis Foundation (NPF) endorse topical vitamin D analogues, like calcipotriene (calcipotriol) and calcitriol, as effective treatments for psoriasis.^47^ This is supported by several clinical trials evaluating topical vitamin D analogue efficacy (Table 1).^64–70^ Although topical corticosteroids are the most common treatment for this condition, they are generally recommended for short-term use (4–8 weeks) given the additional risk of skin atrophy, striae, folliculitis, telangiectasia, tachyphylaxis and purpura. Systemically, corticosteroid usage presents risk for cataracts, glaucoma, adrenal suppression, decreased growth rate, hypertension, hyperglycemia and Cushing syndrome.^71^ The risk of over-­reliance on topical corticosteroids highlights the importance of steroid-sparing agents such as topical vitamin D analogues, which may offer a more effective and sustainable option for long-term, systemic psoriasis treatment. Thus, these agents are recognized as effective and steroid-sparing alternatives for long-term management due to their favourable safety profile, especially in reducing the risk of skin atrophy and other corticosteroid-associated complications. Recently, newer topical agents such as topical aryl hydrocarbon receptor modulators and topical phosphodiesterase-4 inhibitors are increasingly utilized in psoriasis treatment, but may be limited in terms of cost effectiveness.^72^

**Table 1:** Clinical trials of topical vitamin D analogue for psoriasis

Author, year	Participants (*n*)	Study location	Psoriasis subtype	Male (%)	Average age (years)	Duration	Treatment/dosage	Outcomes	Adverse events
Calcipotriol/calcitriol monotherapy									
Kragballe *et al*., 1988^48^	30	Denmark	Chronic plaque-type	30%	39	6 weeks	Calcipotriol cream twice daily (10, 33 or 100 µg/g) Placebo twice daily	Symptom improvement for 10 µg/day in 22% of patients Symptom improvement for 33 µg/day in 56% of patients Symptom improvement for 100 µg/day in 78% of patients	None
Dubertret *et al.*, 1992^49^	66	France	Psoriasis vulgaris	69.7%	43	4 weeks	Calcipotriol ointment twice daily (50 µg/g) Placebo	PASI score fell from 14.2 to 6.3 in calcipotriol-treated patients PASI score fell from 14.1 to 9.2 in placebo	1 patient withdrew due to burning sensation, other adverse events report lesional or perilesional irritation
Ramsay *et al.*, 1994^50^	167	Canada	Chronic plaque-type			52 weeks	Calcipotriol ointment twice daily (50 µg/g)	PASI score decline from 8.1 (SD ± 6.67, *n* = 167) to 2.71 (± 2.12, 98) at 12 months	9 patients withdrew due to irritant reactions
Zhu *et al.*, 2007^51^	250	China	Chronic plaque-type	65.6%	47.1	12 weeks	Twice daily calcitriol ointment (3 µg/g) Twice daily calcipotriol ointment (50 µg/g)	Global improvement score averaged 2.27 for calcitriol and 2.22 for calcipotriol	19 total patients reported adverse events
Calcipotriol combination therapy									
Brands *et al.*, 1999^52^	53	Netherlands	Plaque-type		45	Study ran until lesion clearage, no further improvement seen, or withdrawal reasons	Calcipotriol ointment (50 µg/g) twice daily for test participants (*n* = 25) Low-dose NB-UVB phototherapy 3 times weekly for all participants	Combination therapy showed mean PASI reduction 13.2 to 3.0 (*P* < 0.001) Low-dose NB-UVB showed mean PASI reduction 12.5 to 3.1 (*P* < 0.001)	2 patients withdrew due to skin irritation
Douglas *et al.*, 2002^53^	1106	UK	Psoriasis vulgaris	59.8%	47.1	8 weeks	Twice daily calcipotriol and betamethasone dipropionate ointment (Diprosone^TM^) (*n* = 372), betamethasone dipropionate monotherapy (*n* = 365), or calcipotriol monotherapy (*n* = 369) for the first 4 weeks Twice daily calcipotriol ointment (Daivonex) for the remaining 4 weeks	Mean PASI score after combination treatment was 2.5 Mean PASI score after betamethasone-only was 3.9 Mean PASI score after calcipotriol-only was 4.4	Lesion/perilesional adverse event in combination, betamethasone-only and calcipotriol-only were reported in 8.1%, 4.7% and 12.0% of patients
Guenther *et al.*, 2002^54^	828	Canada	Psoriasis vulgaris	64%	48.5	4 weeks	Calcipotriol 50 µg/g with betamethasone dipropionate ointment 0.5 mg/g once daily Calcipotriol 50 µg/g with betamethasone dipropionate ointment 0.5 mg/g twice daily Calcipotriol ointment 50 µg/g twice daily Vehicle ointment twice daily	PASI reduction in combination therapy once daily was 68.6% PASI reduction in combination therapy twice daily was 73.8% PASI reduction in calcipotriol twice daily was 58.8% PASI reduction in vehicle ointment was 26.6%	Lesional/perilesional adverse reactions reported in 9.9%, 10.6%, 19.8% and 12.5% in combination once daily, combination twice daily, calcipotriol twice daily and vehicle twice daily, respectively
Katoh *et al.*, 2003^55^	61	Japan	Psoriasis vulgaris			6 weeks	0.005% Calcipotriol ointment once daily 0.004% calcipotriol/0.01% clobetasol propionate ointment once daily	Eruption score of truncal regions after 6 weeks was lower in calcipotriol/clobetasol propionate combination therapy (*P* = 0.026)	Fewer adverse reactions were reported in combination therapy cohort
Roussaki-Schulze *et al.*, 2005^56^	45	Greece	Plaque-type	75.6%	48.4	12 weeks	Calcipotriol ointment with UVA1 phototherapy Calcipotriol ointment with NB-UVB phototherapy Calcipotriol ointment monotherapy	PASI score showed statistically significant regression between monotherapy and combination therapy (*P* = 0.027)	None
Thaci *et al.*, 2010^57^	730	15 European countries	Chronic plaque-type	68.6%	45.1	16 weeks	Adalimumab (80 mg week 0 and 40 mg every other week) Calcipotriol/betamethasone dipropionate ointment (once daily for 4 weeks)	PASI 75 was initially higher with combination treatment (14.8% vs 5.8%); no statistical significance at week 16 (*P* = 0.086)	4.2% of patients experienced serious adverse events likely not related to treatment (serious infection, tumorigenesis)
Levine *et al.*, 2010^58^	168	USA	Chronic plaque-type			12 weeks	Randomized to 2 of 7 treatments ointment Placebo Calcipotriene 0.005% alone	50.0% of patients in the calcipotriene and nicotinamide 1.4% combination group achieved a clear to almost clear outcome at week 12	4 patients discontinued
							Nicotinamide 1.4% alone Calcipotriene and nicotinamide 0.05% Calcipotriene and nicotinamide 0.1% Calcipotriene and nicotinamide 0.7% Calcipotriene and nicotinamide 1.4%		
Takahashi *et al.*, 2013^59^	40	Japan	Plaque-type	85%	56.1	12 weeks	Calcipotriol ointment twice daily Calcipotriol ointment twice daily and NB-UVB phototherapy weekly Heparinoid ointment twice daily and NB-UVB phototherapy more than twice a week Calcipotriol ointment twice daily and NB-UVB phototherapy more than twice a week	PASI score decreased the most with calcipotriol ointment twice daily and NB-UVB phototherapy more than twice a week Psoriasis disability index significantly decreased (*P* < 0.01)	None
Bagel *et al.*, 2020^60^	50	USA	Plaque-type			16 weeks	Oral apremilast (30 mg twice dialy) for first 8 weeks Daily calcipotriene/betamethasone dipropionate foam (0.005%/0.64%) added on in partial responders to apremilast	26 patients were partial respondents to apremilast and candidates for combination therapy. 21/26 patients on combination therapy achieved PASI 75 at week 12, PGA improved by 67%, BSA improved by 86% 8 patients achieved PASI 75 at week 8 on apremilast monotherapy Apremilast non-responders were discontinued at week 8	>5% occurrence due to headache, diarrhoea and nausea
Lebwohl *et al.*, 2021^61^	251	USA	Truncal and limb lesions	65.2%	51.8	52 weeks	Twice weekly calcipotriene 0.005%/betamethasone dipropionate 0.064% (Cal/BD) foam Twice weekly vehicle foam	Days of remission for treatment group was 41 days longer than vehicle foam-controlled group (*P* < 0.001)	3 patients (2 in proactive and 1 in reactive group) withdrew due to adverse events
Calcipotriol formulations									
Koo *et al.*, 2016^62^	376	USA	Psoriasis vulgaris	52.7%	51.3	4 weeks	Once daily Cal/BD aerosol foam (0.005%) Once daily Cal/BD ointment (0.064%) Once daily vehicle foam Once daily vehicle ointment	Mean difference in PASI score was −0.6; *P* = 0.005 between aerosol foam and ointment	1 adverse drug reaction with foam application
Paul *et al.*, 2017^63^	463	France	Psoriasis vulgaris	63.7%	54.1	8 weeks	Once daily calcipotriol 50 µg/g (Cal) plus betamethasone 0.5 mg/g (BD) foam Once daily calcipotriol 50 µg/g (Cal) plus betamethasone 0.5 mg/g (BD) gel Once daily vehicle foam Once daily vehicle gel	PASI 75 was achieved by 52% of foam treatment users by week 4 compared with 32% of gel users at week 8	14 adverse reactions in foam application and 7 with gel application

BD, betamethasone dipropionate; BSA, body surface area; Cal, calcipotriene; NB-UVB, narrowband UVB; PASI, Psoriasis Area and Severity Index.

### Calcipotriene

As a monotherapy, topical calcipotriene demonstrates consistent efficacy in the treatment of mild-to-moderate plaque-type psoriasis, with significant improvements in Psoriasis Area and Severity Index (PASI) scores.^73^ Short-term clinical trials have demonstrated the reliability of calcipotriene as a psoriasis treatment. A double-blind trial from Denmark of 10, 33 and 100 µg/g twice-daily treatment of calcipotriol cream for 6 weeks resulted in 22%, 56% and 78% of patients, respectively, reporting symptom improvement.^48^ Long-term effects of calcipotriol treatment were observed in a 52-week, multicentre Canadian clinical trial that included 167 adults with chronic plaque psoriasis who had previously responded to calcipotriol. In this study, patients applied calcipotriol ointment (50 μg g^−1^) twice daily until remission was achieved.^50^ At the 12-month follow-up, 26% of patients had complete clearing and the rest required continuous treatment despite a significant decrease in PASI score from 8.1 (SD 6.67, *n* = 167) at baseline to 3.90 (SD 3.50, *n* = 136) at 2 months and 2.71 (SD 2.12, *n* = 98) at 12 months. These studies highlight calcipotriene’s consistency and effectiveness for treating mild to moderate plaque-type psoriasis as a monotherapy. Long-term studies also indicate a substantial portion of patients achieve complete clearing, highlighting its potential as a mainstay treatment.

### Calcitriol

Topical calcitriol has only been used in the ointment form. Zhu *et al*. conducted a 12-week trial of calcipotriol ointment and calcitriol ointment treatments for chronic plaque-type psoriasis and found similar efficacy of both.^51^ Calcitriol 3 µg g^−1^ twice daily for up to 8 weeks has been suggested as a potential treatment option with less severe skin irritation.^70^

### Combination therapies

#### Topical vitamin D analogues and ­corticosteroids

Combination therapy treatments for psoriasis are reliable options for long-term treatment, demonstrating enhanced treatment effectiveness and longer periods of remission.^58,74^ Combination therapy with different agents (topical corticosteroids, phototherapy, biologics) can either enhance or maximize the treatment efficacy and minimize risks or adverse events of long-term medication use. Many studies using vitamin D in an ointment form have analysed the effectiveness of calcipotriol/betamethasone (C/B) treatment compared with the standalone corticosteroid, finding a greater change in PASI score in the combination group. In a randomized controlled trial assessing calcipotriol/betamethasone combination therapy (Daivobet^TM^), patients who used the combination ointment (containing 50 µg g^−1^ of calcipotriol and 0.5 mg g^−1^ of betamethasone) once or twice daily experienced significant improvements in psoriasis severity. Another study with adalimumab combined with C/B ointment demonstrated that C/B ointment, applied once daily, led to rapid improvements in PASI scores in the initial 4 weeks compared with the control group. After the first 4 weeks, adalimumab monotherapy showed a slight edge in PASI improvement, but the combination therapy had a stronger initial impact. In a large-scale, double-blind trial, C/B ointment applied twice daily showed a significantly greater reduction in PASI compared with either calcipotriol or betamethasone alone, with a rapid onset of action. This combination ointment was well tolerated, with fewer adverse effects compared with the individual treatments, demonstrating its efficacy and safety as a preferred ­therapy.^53,57,75–^77^^

In a few studies, C/B outperformed calcipotriol and/or betamethasone monotherapy.^61,78^ This combination treatment with calcipotriol has been shown to be well tolerated as a long-term protocol, observing 41 more remission days for the calcipotriene 0.005%/betamethasone dipropionate 0.064% (Cal/BD) foam treatment group compared with the vehicle foam control group (*P* < 0.001).^61^ A meta-­analysis of vitamin D analogues and various corticosteroids revealed that when compared with vitamin D derivative monotherapy, the combination treatment led to a 22% increase in the likelihood of clearance.^79^ When compared with corticosteroid monotherapy, the combination treatment led to a 20% increase in the clearance likelihood. Furthermore, significant differences between corticosteroid classes and likelihood of disease clearance were found (*P* = 0.04). Combining topical vitamin D analogues with corticosteroids, particularly calcipotriol with betamethasone, has shown to enhance treatment effectiveness and extend periods of remission. This synergy suggests a robust strategy for improving clinical outcomes and patient quality of life.

#### Topical vitamin D analogues and phototherapy.

Calcipotriol with UVB phototherapy has also been studied in conjunction with the majority of protocols utilizing calcipotriol twice daily and UVB for twice to thrice a week.^52,56,59,79^ Studies from Greece and Japan both reported a significant benefit to narrowband (NB)-UVB more than twice a week with 50 µg/g of calcipotriol twice daily as opposed to the UVB monotherapy.^56,59^ Conversely, a single-blind, controlled study from the Netherlands showed no significant add-on effect with calcipotriol ointment and NB-UVB therapy among the 53 enrolled patients.^52^ A meta-analysis of four studies found no significant difference between UVB combination therapy and UVB monotherapy (11% increased likelihood of clearance, *P* = 0.07).^79^ A major limitation to these studies involves concurrent sun exposure that may confound with UVB light therapy protocol. In addition, psoriasis scale thickness may contribute to diminished UV effect.

#### Topical vitamin D analogues and other oral medications.

Combination treatments with other medications including biologics such as adalimumab or other oral medications like apremilast or nicotinamide have been less studied.^57,58,60^ In a single-centre US study done with apremilast and Cal/BD foam topical therapy, partial respondents (PASI 25–PASI 75) to 8-week apremilast therapy added on Cal/BD topical therapy until week 16. Most of these patients reached PASI 75 at week 16, while 20% of patients on apremilast monotherapy reached PASI 75.^60^ With adalimumab, a 730 patient European study showed combination with Cal/BD and adalimumab to be more rapid and higher efficacy at week 4 compared with adalimumab alone.^57^ However, subsequent weeks showed adalimumab monotherapy to be more effective. A Ukrainian pilot study of 164 patients with calcipotriene and nicotinamide combination showed 50% clearing of psoriasis lesions at week 12, while other standalone treatments (placebo, nicotinamide-only and calcipotriene-only) were at 18.8%, 25.0% and 31.5%, respectively.^58^ To validate these studies, multicentre, double-blind studies that also address dose dependency are necessary to understand the use of a topical vitamin D analogue and a systemic drug or other oral medications like nicotinamide to quickly improve psoriatic flares and manage a stable psoriatic disease course.

### Topical vitamin D analogue formulations and drug delivery systems.

Advances to drug formulation have been made to increase efficacy of drug delivery.^80^ In addition, drug formulation may play roles in patient adherence, which directly affects drug efficacy.^81^ Comparison studies between available formulations (ointment, gel and foam) have been conducted. A multicentre double-blind phase II study was conducted over 376 patients comparing the Cal/BD foam and ointment at the endpoint determined by the Physicians’ Global Assessment (PGA) score.^62^ At 4 weeks, the foam and ointment deliverables, respectively, had 54.6% and 43.0% patients reach the endpoint and the mPASI (modification of PASI) assessment favoured the foam formulation significantly at both 1- and 4-week timepoints. This was supported by other randomized, double-blind, vehicle-controlled studies that reported calcipotriol ointment as effective in decreasing PASI, but significantly lower than combination formulations such as Cal/BD foam not accounting for difference in drug formulations.^49,54,55,82^ The PSO-ABLE study comparing the foam and gel Cal/BD formulations of 463 patients showed a significantly higher percentage of patients with foam Cal/BD to reach therapeutic success in 4 weeks as opposed to gel Cal/BD.^63^ In addition, quality of life measures were higher in patients using the foam formulation compared with gel.^83^ This suggests that the ease of use and acceptability of the foam may enhance patient adherence, which is crucial for achieving sustained high efficacy rates.

Recent work has investigated different drug delivery systems that are more effective and satisfactory for patients. These drug delivery systems may be applied to topical vitamin D treatments to improve efficacy and patient adherence. Innovation regarding nano-technology delivery systems such as polyaphron dispersion technology has promise in improving penetration, local tolerability and ease of application.^84^ These systems create multimolecular surfactant layers that embed oil-based drug molecules and suspend them into polymeric gel. This design allows flexibility in concentration, polymeric molecular weight and potential synergistic interactions, enabling specificity in targeting relevant body locations and improvement in tolerability of the drug formulation. Another area of discovery is the usage of microneedle patches as a delivery system.^85^ Advances in drug delivery systems, such as foam-based vehicles and nanoparticle technologies, have enhanced the efficacy and quality of life for patients using topical vitamin D treatments, possibly in part due to more favourable formulations leading to increased patient adherence. As such, studying poor adherence in relation to drug efficacy for these topical treatments remains under-addressed, and may significantly impact the overall effectiveness of these products.

Overall, topical vitamin D is an effective treatment for psoriasis. The pairing of topical vitamin D with other treatments like betamethasone suggests increased efficacy and prolonged remission time. Furthermore, formulation of drug delivery systems, particularly foam-based prescriptions, may offer differential treatment outcomes. Discussion of efficacy particularly on the standardization of dosing that differs on formulation (i.e. foams are more standardized while creams and ointments depend on patient discretion) may require investigation as a limitation to the above studies.

#### Oral vitamin D treatment

The biological foundation for vitamin D’s effectiveness for psoriasis treatment is well established, leading to the inclusion of topical vitamin D analogues in modern treatment options. However, oral vitamin D supplementation as a treatment for psoriasis has been less studied and is not currently recommended as a treatment option by the joint AAD and NPF.^47^ Some clinical studies have shown promising effects of oral vitamin D on psoriasis management, but high dosing strategies cited by those works present risk of hypercalcaemia or hypercalciuria. While oral vitamin D’s role in psoriasis treatment is less clear, emerging evidence points to its potential benefits, especially in patients with low serum vitamin D levels. However, the risk of hypercalcemia with high-dose supplementation necessitates further investigation to establish safe and effective dosing protocols.

### Circulating vitamin D levels in patients with psoriasis

A healthy population should observe serum vitamin D levels of about 20–50 ng/mL.^86^ Hypovitaminosis D, specified as serum levels <20 ng/mL, is associated with higher risk of immune system events, cardiovascular events and maternal health complications.^87^ Specific to psoriasis, a meta-­analysis of prospective cohort, retrospective cohort, case–control and cross-sectional studies in English and a recent cross-sectional study reported that patients with psoriasis had significantly lower serum vitamin D levels than those without psoriasis.^88–90^ In one of the case–­control studies conducted in Spain, the prevalence of vitamin D deficiency, defined as <20 ng/mL, in patients with psoriasis was 25.6%, compared with 9.3% of controls without psoriasis.^91^ Furthermore, retrospective cross sectional and case–control studies from Brazil, Iran and Italy have shown a negative correlation between vitamin D levels and psoriasis severity.^90,92,93^ This suggests that vitamin D deficiency could be a risk factor for the pathogenesis or incidence of psoriasis as well as progression of psoriasis. Conversely, a US population-wide sampling study using National Health and Nutrition Examination Survey data did not find significance between vitamin D deficiency levels in psoriatic and control groups.^94^ Body surface area involvement with psoriasis was not significantly associated with vitamin D level either. Potential confounders include seasonal variation of vitamin D status and BMI.^95,96^ Because vitamin D can be synthetized endogenously with the influence of UVB radiation, seasonal exposure to sunlight can improve vitamin D status.^97,98^ The effect of seasonal variation on disease activity or improvement is not clear, which necessitates further study. In addition, BMI is positively associated with psoriasis and negatively associated with vitamin D levels, presenting a potential confounder for vitamin D status in studies of psoriasis.^91,94^

### Clinical trials of oral vitamin D supplementation and psoriasis

The early clinical trials for oral vitamin D were conducted from 1986 to 1996 to determine the effects of oral vitamin D on improvement of lesions in general plaque-type psoriasis patients. Studies were mostly small-scale (<50 participants), open-label trials, of which short-term (2–6 months) daily supplementation of was tested (Table 2). Given that the recommended dietary allowance of vitamin D (calcitriol) supplementation is 600 IU/day or 15 µg/day, these trials only involved small doses (0.5–1 µg/day) of calcitriol [1,25(OH)2-D3,1,alpha 25-dihidroxyvitamin D], which is the active form of vitamin D used in systemic therapies, or alphacalcidol [1α-(OH)D3] supplementation.^113^ In contrast, calcipotriene (a vitamin D analogue) is typically used in topical formulations for psoriasis treatment and is not commonly used in systemic therapies. Only one of the eight clinical trials used a placebo-controlled design. This Saudi Arabian placebo-controlled double-blind study of 50 patients found no significant difference with 45% and 38%, respectively, of calcitriol and placebo treatment groups experiencing slight improvement in psoriatic symptoms in the calcitriol group.^103^ The other seven studies, including a few US studies, found that daily oral vitamin D supplementation at a dose of 0.5–1.0 µg/day led to a decrease in psoriatic symptoms, determined through different outcomes such as psoriasis lesion coverage, PASI scores or self-reported indications of psoriasis severity.^99–102,104,106^

**Table 2:** Clinical trials of oral vitamin D for psoriasis

Study	Participants (*n*)	Study location	Psoriasis subtype	Male (%)	Average age (years)	Duration	Treatment/dosage	Outcome metrics	Adverse events
Takamoto *et al.*, 1986^99^	7	Japan	Psoriasis vulgaris	85.7%	53.6	12 months	1.0 μg/day 1α-(OH)D3	4 had complete or almost complete remission 2 patients had minimal improvement	None
Smith *et al.*, 1988^100^	14	USA	Moderate to severe psoriasis vulgaris			2 months	0.25 µg once or twice/day 1,25(OH)2-D3 Increased in 0.25 and 0.5 µg intervals until 2.0 µg/day	77% remaining patients had >50% clearing	2 cases of persistent hypercalciuria
Morimoto *et al.*, 1989^101^	40	Japan	Psoriasis vulgaris	—	44	6 months	1.0µg/d 1,25(OH)2-D3 0.5 µg/day 1α-(OH)D3	76% of patients with 1,25(OH)2-D3 had moderate improvement or higher 25% of patients with 1α-(OH)D3 showed a moderate improvement	None
Holland *et al.*, 1989^102^	15	UK	Plaque-type			4–6 months	1.0 µg/day 1α-(OH)D3	7 patients demonstrated complete resolution 3 patients demonstrated partial resolution 5 patients had a lack of response	None
Siddiqui *et al.*, 1990^103^	50	Saudi Arabia	Psoriasis vulgaris			3 months	1.0 µg/day 1α-(OH)D3 Daily placebo	45% 1α-(OH)D3 patients had slight improvement 38% control patients had slight improvement	None
Lugo-Somolinos *et al.*, 1990^104^	10	Puerto Rico	Moderate to severe psoriasis vulgaris			3 months	0.5 µg/day 1,25(OH)2-D3	4 cases showed moderate improvement	None
el-Azhary *et al.*, 1993^105^	8	USA	Psoriasis vulgaris			6 months	0.5 µg/day 1,25(OH)2-D3 Increased 0.5 µg biweekly until max 2.0 µg/day 1,25(OH)2-D3	12.5% patients had major improvement 12.5% patients had moderate improvement 75% patients had little to no improvement	None
Perez *et al.*, 1996^106^	85	USA	Psoriasis vulgaris	72.9%	46	24 months	0.5 µg/day 1,25(OH)2-D3 Increased 0.5 µg biweekly	PASI baseline changed from 18.4 to 7.8 after 24 months of treatment	None
Finamor *et al.*, 2013^107^	9	Hungary	Psoriasis vulgaris	55.6%	45.3	6 months	35 000 IU daily vitamin D3	PASI score significantly improved in all 9 patients and vitamin D status increased from 4.9 ± 7.4 to 106.3 ± 31.9 ng/mL (*P* = 0.0011)	None
Ingram *et al.*, 2018^108^	101	New Zealand	Chronic plaque-type	55.4%	49.4	12 months	100 000 IU monthly vitamin D3 Placebo	PASI score did not differ significantly between severity grouping (*P* = 0.49 and *P* = 0.90); vitamin D serum levels increased for both groups	None
Jarret *et al.*, 2018^109^	65	USA	Not specified	61.5%	66	12 months	100 000 IU monthly vitamin D3 Placebo	PASI, PGA, PDI and DLQI scores were not significantly different between supplementation and placebo groups (*P* > 0.05)	None
Disphanurat *et al.*, 2019^110^	45	Thailand	Chronic plaque-type	53.3%	50.9	6 months	60 000 IU/2 weeks vitamin D2	Mean PASI score improvement in treatment group was 42.79 ± 3.62% Mean PASI score improvement in placebo group was 21.57 ± 53.22%	Reported drowsiness and nausea in treatment and placebo groups
Hahn *et al.*, 2022^111^	278	USA	Not specified	49.4%	67.1	5.3 years median follow-up	2000 IU/day vitamin D3 Placebo	Daily vitamin D supplementation resulted in a hazard ratio of 0.72 with *P* = 0.27	Low number of side effects reported
Jenssen *et al.*, 2023^112^	122	Norway	Plaque-type	62.2%	53.7	4 months	100 000 IU initial and 20 000 IU/week vitamin D3	No significant difference on psoriasis severity (*P* = 0.52)	None

1α(OH)D3, alphacalcidol; 1,25(OH)2-D3, calcitriol; DLQI, Dermatology Life Quality Index; PASI, Psoriasis Area and Severity Index; PGA, Physicians’ Global Assessment; PDI, Psoriasis Disability Index.

Later studies involved long-term studies (4–12 months), generally larger study sizes (<100) and included open-label trials and placebo-controlled double-blind trials (Table 2). Two double-blind, placebo-controlled randomized trials found no benefit to vitamin D supplementation. In their study, Ingram *et al*.^108^ recruited 101 participants with psoriasis and randomized to receive either 100 000 IU/month of oral vitamin D3 for 12 months or a placebo. The primary outcome, assessed using PASI, showed no significant difference between the vitamin D and placebo groups at any of the follow-up points [group F(1, 104) = 0.48, *P* = 0.49; group*time F(4, 384) = 0.26, *P* = 0.90]. Despite this, serum levels of 25(OH)D increased in both groups over time. A post hoc analysis that higher serum 25(OH)D (up to 125 nmol/L) was associated with mild decreases in PASI scores (estimated decrease: 0–2.6, *P* = 0.002). Another large-scale study by Jarrett *et al*.^109^ which investigated the clinical effect of oral vitamin D3 supplementation, also found no significant difference in psoriasis outcomes between the vitamin D3 and placebo groups after 12 months. In this study, 23 participants received 100 000 IU of vitamin D3 monthly, while 42 others received a placebo. No significant differences were observed between the groups in terms of PASI, PGA, Dermatology Life Quality Index (DLQI) or Psoriasis Disability Index (PDI) scores at the 12-month mark. These results further support that vitamin D supplementation alone may not result in significant improvement in psoriasis severity.^108,109^ In more recent years, Hahn *et al*. reported results of the Vitamin D and marine omega-3 fatty Acid (VITAL) randomized controlled trial, a trial of over 25 000 adults testing the effects of oral vitamin D supplementation (2000 IU/day) and/or marine-derived long-chain omega 3 fatty acids on risk of all autoimmune diseases compared with a placebo. Specific to psoriasis incidence, 2000 IU/day of cholecalciferol resulted in a hazard ratio of 0.72 (95% confidence interval 0.39–1.37; *P* = 0.27).^111^ Interestingly, in the 2-year follow-up study after randomized supplementation was concluded, the risk of developing psoriasis trial start through a mean of 7.3 years had fallen to 0.61 (0.38–0.98, *P* = 0.04).^96^ These results suggest supplementation may be protective against developing psoriasis long term. In 2023, a Norwegian randomized, double-blind placebo-controlled clinical trial was conducted with 122 psoriasis patients using oral cholecalciferol at a 20 000 IU/week dose, adjusting for seasonal variation. No significant changes to the PASI, PGA or the DLQI scores were found.^112^

These studies on oral vitamin D report differential outcomes and exhibit study limitations. Earlier trials generally employed minimal doses of the active form vitamin D (calcitriol), yet still observed some positive impacts, some of which cite significant resolution in psoriasis lesion coverage. Later studies found less conclusive evidence supporting the benefits of oral vitamin D (cholecalciferol) on psoriasis. Although current data are promising, there remains a need for larger, multicentre, double-blind clinical trials to ascertain the full therapeutic potential of oral vitamin D in psoriasis and to address the evidence gaps, particularly in the context of oral supplementation and combination therapies. Furthermore, there is variability in the response to different forms of vitamin D, with some studies indicating superior outcomes with calcitriol over calcipotriene. This necessitates further head-to-head comparisons to optimize treatment regimens.

### Oral vitamin D combination therapy

The past clinical trials solely observed the change in psoriasis outcome based on vitamin D supplementation. The studies controlled for compounding treatment effect by only including patients of with stable psoriasis and maintaining all patients on their current treatments preclinical trial. However, the effect of combined therapies with oral supplementation may improve efficacy of monotherapy. Limited studies have been done to observe the effects of oral vitamin D as a combination therapy to improve psoriasis outcome and long-term remission of psoriasis. Options including combination therapy with NB-UVB and acitretin had varying outcomes.^114–116^ Generally, using combination therapy with vitamin D supplementation increased improvement of PASI scores. However, long-term studies are still needed to support study results.

Overall, the usage of oral vitamin D is promising, but requires further study. Additional studies should focus on the effects of oral vitamin D monotherapy with dose ­dependency in consideration and incorporating additional vitamin D supplementation to pre-existing treatments as a combination therapy.

Many clinical studies and trials have examined the efficacy of vitamin D in the treatment of psoriasis, using both topical and oral preparations. Topical vitamin D analogues are recognized as effective for long-term management, particularly when used in combination with other psoriasis therapies like betamethasone. Further modifications to topical drug formulations also demonstrate differential efficacy, with foam-based formulations improving psoriasis clearance and quality of life. In addition, recent developments in newer topicals such as topical aryl hydrocarbon receptor modulators and topical phosphodiesterase-4 inhibitors may be increasingly utilized, but given current cost-effectiveness, topical vitamin D may still be optimal. Oral vitamin D’s role in psoriasis treatment is less clear. However, emerging evidence shows potential benefits, especially regarding combination therapies and usage among patients with low circulating vitamin D levels. Limitations including the lack of extensive, multicentre, double-blind clinical trials and a standardized oral vitamin D treatment protocol exist. To fully determine the therapeutic potential of vitamin D in treating or preventing psoriasis, future trials should specifically address evidence gaps, especially in the realm of oral supplementation and combination therapies, so that we may better understand the role of vitamin D in psoriasis prevention and treatment and provide definitive, practical guidelines for clinicians and patients.

## Data Availability

There are no new data associated with this article.
